# Stereotactic Body Radiation Therapy for Recurrent, Isolated Hepatocellular Carcinoma Lymph Node Metastasis With or Without Prior Liver Transplantation

**DOI:** 10.7759/cureus.9988

**Published:** 2020-08-24

**Authors:** Tyler Walburn, Andrew M Moon, Paul H Hayashi, David Gerber, Hanna K Sanoff, Katrina A McGinty, David Mauro, Joel Tepper, Kyle Wang

**Affiliations:** 1 Department of Radiation Oncology, University of North Carolina at Chapel Hill School of Medicine, Chapel Hill, USA; 2 Division of Gastroenterology and Hepatology, University of North Carolina at Chapel Hill School of Medicine, Chapel Hill, USA; 3 Division of Abdominal Transplant, University of North Carolina at Chapel Hill School of Medicine, Chapel Hill, USA; 4 Division of Hematology and Oncology, University of North Carolina at Chapel Hill School of Medicine, Chapel Hill, USA; 5 Department of Radiology, University of North Carolina at Chapel Hill School of Medicine, Chapel Hill, USA; 6 Division of Vascular Interventional Radiology, University of North Carolina at Chapel Hill School of Medicine, Chapel Hill, USA

**Keywords:** hepatocellular carcinoma, lymph node metastases, stereotactic body radiation therapy, liver transplantation

## Abstract

Lymph node metastases from hepatocellular carcinoma (HCC) represents a challenging clinical scenario with a poor prognosis, especially in the setting of prior liver transplant. Long-term survival is achievable in select patients with isolated lymph node metastases who undergo surgical resection, but little data exist regarding non-surgical options. For intrahepatic HCC, stereotactic body radiation therapy (SBRT) has emerged as a standard and effective nonsurgical treatment option. Here, we present three patients (two with prior liver transplant) with isolated lymph node metastases treated with curative intent using SBRT to doses of 30-45 Gy in three to five fractions. Two patients (with follow-up of 27 and 31 months) had a complete or near-complete response and remain cancer-free. One patient had intrahepatic HCC recurrence shortly after SBRT but stable disease in the treated lymph node metastasis at 20 months. Liver function remained excellent after radiation in all three patients, but one patient developed a grade 3 duodenal ulcer at 20 months that resolved with medical management. These cases illustrate the potential utility of SBRT as a non-invasive, definitive treatment option for patients with isolated lymph node metastases from HCC.

## Introduction

Lymph node metastases (LNM) occur in approximately half of hepatocellular carcinoma (HCC) patients with extrahepatic spread [1], and these patients have a median survival of less than one year [1]. In patients with a history of a liver transplant, recurrent HCC portends an especially poor prognosis. Nonetheless, in appropriately selected patients who have isolated LNM (i.e. no other sites of disease), there are reports of surgical resection leading to long-term survival [2]. However, many patients are not candidates for surgery and little data exist regarding effective non-surgical options. The purpose of this case series is to report the capability of stereotactic body radiation (SBRT) to achieve similar long-term results in patients with isolated HCC LNM.

SBRT has recently been established as a standard and effective treatment option for nonsurgical localized intrahepatic HCC, reflected by its inclusion in the 2019 National Comprehensive Cancer Network Guidelines and practice guidance statement from the American Association for the Study of Liver Diseases [3,4]. With local control rates of 70-80% even for large tumors, SBRT demonstrates equivalent and potentially superior safety and efficacy compared to other liver-directed treatments such as trans-arterial chemoembolization (TACE) or thermal ablation, provided that radiation dose to surrounding hepatic tissue is minimized and pre-treatment liver function is taken into consideration [5-7]. Though the role of radiation therapy for LNM from HCC has traditionally been minimal and limited to palliation, SBRT allows for the precise delivery of ablative radiation doses in three to five treatment sessions with the potential of long-term disease control. We herein present a series of three patients treated using SBRT with curative intent for isolated, recurrent LNM from HCC, including two patients with a history of liver transplantation.

## Materials and methods

We performed a retrospective study of patients with isolated LNM from HCC treated with SBRT at our institution from 2016 to 2019. Patients with active intrahepatic disease were excluded. Treatment responses were defined based on Response Evaluation Criteria in Solid Tumours (RECIST): complete response = disappearance of target lesion or reduction in short axis of a pathologic lymph node to <10 mm; partial response = 30% reduction in sum of diameters; progressive disease = 20% increase in the sum of diameters; stable disease = reduction or increase in size that does not meet criteria for partial response or progressive disease [8].

## Results

Clinical and treatment characteristics for patients A, B, and C are shown in Table 1. Patients A and C had a history of liver transplant. Follow-up was 20, 27, and 31 months after SBRT for patients A, B, and C respectively, and all patients are alive at the time of this report. Patients B and C remain cancer-free, whereas patient A had multifocal recurrence in the liver at two months, but stable disease at the irradiated LNM at 20 months.

**Table 1:** Summary of three patient cases

Table 1			
	Patient A	Patient B	Patient C
Baseline characteristics			
Age (years)	63	74	68
Etiology of cirrhosis	Hepatitis C	Alcohol	Hepatitis C
Liver transplant	Yes	No	Yes
Prior liver-directed treatments	TACE, ablation	TACE, ablation	ablation
Recurrent LNM diagnosis	Biopsy	MRI	MRI
LNM size	3.6 x 3.3 cm	6.4 x 3.9 cm	2.3 x 1.8 cm
AFP	3	n/a	1130
Total bilirubin	0.3	0.7	0.4
Albumin	4.1	4.2	4.4
INR	1.1	1.1	1.2
Child-Pugh	A	A	A
Treatment and outcomes			
Follow-up after SBRT	1Y, 8M	2Y, 3M	2Y, 7M
SBRT modality	Cyberknife	Linear accelerator	Cyberknife
SBRT dose and schedule	30 Gy in 5 fx (BED 48 Gy), QOD	40 Gy in 5 fx (BED 72 Gy), QOD	45 Gy in 3 fx (BED 113 Gy), QOD
Status of treated LNM	Stable disease	Near-complete response (after 3 months)	Complete response (after 11 months)
Status of HCC	Recurrence in liver at 2 months	No evidence of disease (at 27 months)	No evidence of disease (at 31 months)
Toxicities of SBRT	G1 acute nausea G1 acute fatigue	G2 acute duodenitis G3 late duodenal ulcer	None
Abbreviations: HCC, hepatocellular carcinoma; TACE, trans-arterial chemoembolization; LNM, lymph node metastasis; SBRT, stereotactic body radiation therapy; QOD, every other day; BED, biologically effective dose.			

**Figure 1:**
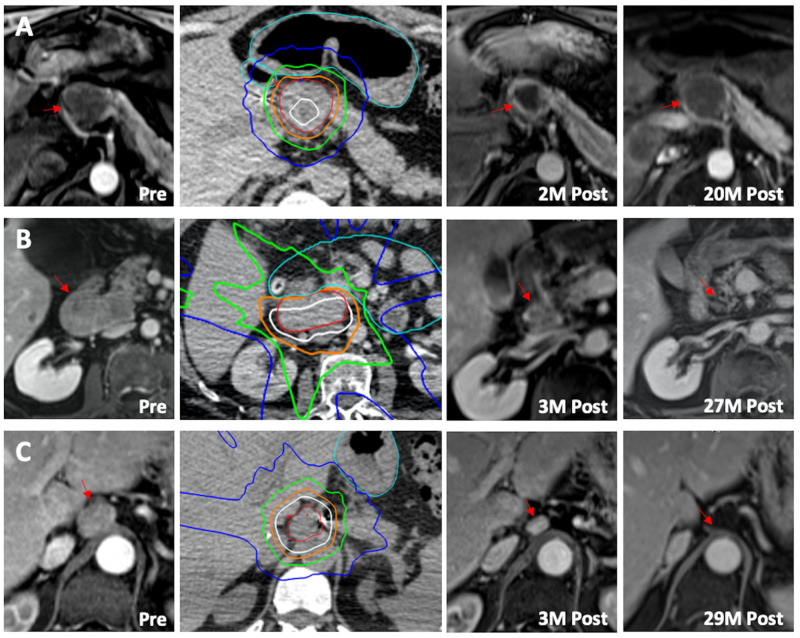
Radiographic response to SBRT SBRT treatment in three patients (A, B, and C) with isolated LNM from HCC, with pre-treatment MRI (1st column, red asterisk = LNM), treatment planning scan (2nd column, red outline = gross tumor volume, light blue = bowel avoidance structure, dark blue = 10 Gy, green = 20 Gy, orange = 30 Gy, white = 40 Gy), initial post-treatment MRI (3rd column), and long-term follow-up MRI (4th column). Patient A had recurrence in the liver at two months and stable disease in the treated node at 20 months, whereas patients B and C remained without evidence of disease recurrence at 27 months and 29 months, respectively.

Figure 1 shows the pre- and post-treatment contrasted MRIs for each patient, along with their radiation plans and dose distributions. Doses are described both as physical dose and with a biologically effective dose (BED) conversion using an alpha/beta of 10 (a mathematical adjustment used to provide an estimate of the relative biological efficacy of varying dose regimens). Doses were reported for both the gross tumor volume (GTV) and planning target volume (PTV, defined as GTV plus a 5-8 mm margin). Critical bowel was defined as the stomach plus duodenum and any other portions of bowel near the target.

Patient A (Figure 1A)

A 63-year-old male with a history of hepatitis C-associated cirrhosis and liver transplantation for HCC presented in 2018 with an enlarging peripancreatic node. He was originally diagnosed with HCC in 2011 and relapsed after treatment with ablation and TACE. He ultimately underwent transplant in 2012 showing two foci of viable HCC on explant pathology. He remained cancer-free on tacrolimus and mycophenolate until 2017, when surveillance MRI showed an isolated peripancreatic lymph node measuring 3.6 x 3.3 cm. Alpha-fetoprotein (AFP) was normal, and biopsy showed hepatocellular carcinoma. Given abutment of the celiac artery, the multidisciplinary tumor board thought that surgical resection would be morbid and recommended radiation with curative intent. He was treated with CyberKnife SBRT using fiducial-tracking to 30 Gy in five fractions (BED 48 Gy), every other day. Mean liver dose was 3 Gy and maximum critical bowel dose was limited to 29 Gy, with 100% of the GTV and 95% of the PTV receiving the full prescription dose of 30 Gy. The patient tolerated radiation treatment well with no late toxicity or change in liver function. He continued to take tacrolimus but mycophenolate was discontinued. Imaging at two months showed slight shrinkage of the treated LNM, but multifocal liver recurrence which was treated with sorafenib. The treated LNM had a partial response (33% reduction in the short axis diameter) at four months, with most recent imaging at 20 months showing slight interval growth (mostly from increasing central necrosis) not meeting criteria for progression. This may have reflected a response to cabozantinib, given concomitant decreasing AFP and size of the patient's parenchymal liver lesions.

Patient B (Figure 1B)

 A 74-year-old male with a history of alcohol-associated cirrhosis and recurrent HCC presented in 2017 with an enlarging portal node. He had been diagnosed with a solitary 5 cm HCC in 2014 and underwent TACE, followed by ablation of a second liver tumor and a pericardial node in 2015. At the time of ablation, he was also found to have an enlarged 3 cm portal node associated with mild epigastric pain, radiographically consistent with HCC. This lesion was originally untreated because of location, and in 2017 had enlarged to 6.4 x 3.9 cm. Staging was completed with MRI including the abdomen and pelvis; lung staging was not performed. The patient then received SBRT on a linear accelerator without fiducials (daily cone beam CT was used for image guidance). We treated the majority of the tumor to 40 Gy in five fractions (BED 72 Gy), every other day, accepting under-dosing of regions adjacent to critical bowel. Mean liver dose was 6 Gy and maximum critical bowel dose was 31 Gy. The volume of bowel receiving 30 Gy (V30) was 0.8 cc, and V25 was 41 cc; 85% of the GTV and 75% of the PTV received the full prescription dose of 40 Gy. Imaging at three months showed an excellent near-complete response, and most recent scans at 27 months showed no evidence of disease recurrence. The patient experienced acute grade 2 duodenitis which resolved after starting pantoprazole. However, 20 months after SBRT and five months after discontinuation of pantoprazole he was hospitalized for symptoms of gastric outlet obstruction and found to have a radiation-associated duodenal ulcer causing duodenal stricture. Symptoms resolved after restarting pantoprazole and sucralfate. Repeat endoscopy showed resolution of the duodenal ulcer. Liver function remained normal after radiation.

Patient C (Figure 1C)

A 68-year-old male with a history of combined kidney/liver transplant for cirrhosis, HCC, and renal failure presented in 2017 with an increasing AFP and an enlarging 2.3 x 1.8 cm celiac lymph node. He had hepatitis C-associated cirrhosis and end-stage renal disease due to diabetic nephropathy. He had originally been diagnosed with a solitary HCC in 2015 and underwent ablation. He then underwent repeat ablation in 2016 for local recurrence, followed by combined kidney and liver transplant. Explanted liver showed a 4.5 cm viable residual HCC. One year post-transplant, the patient presented with an AFP of 1130 (increased from 13 initially post-transplant) and an enlarging, solitary 2.3 x 1.8 cm celiac lymph node consistent with recurrent HCC. He was treated with CyberKnife SBRT using fiducial-tracking to a dose of 45 Gy in three fractions (BED 113 Gy), every other day. Mean liver dose was 5 Gy and maximum critical bowel dose was 15 Gy, with 100% of the GTV and 95% of the PTV receiving the full prescription dose of 45 Gy. The patient tolerated radiation well with no acute or late side effects and no impact on liver function. Imaging at three months showed significant shrinkage of the treated LNM along with a decrease of AFP from 1130 to 11, and a complete response was achieved at 11 months. The patient continued to do well on both tacrolimus and mycophenolate with normalization of AFP and no evidence of recurrence at 31 months.

## Discussion

Here we present three patients treated using SBRT with curative intent for isolated LNM from HCC, two with a history of liver transplant. Two of three patients remain cancer-free with over two years follow-up after SBRT. This is the first report to our knowledge that describes long-term disease control in patients receiving SBRT for solitary LNM from HCC, and supports further investigation of SBRT as a definitive treatment option for LNM. These data are also consistent with the rapidly increasing acceptance of the concept of “oligometastatic” disease for many cancers, a situation where patients with a limited number of metastatic sites are treated with curative intent using serial locoregional therapies such as surgery and SBRT [9,10].

Management of HCC LNM represents a therapeutic challenge, especially when treatment options must be balanced against immunosuppression [11,12]. Although LNM is considered advanced disease (American Joint Committee on Cancer Stage 4, Barcelona Stage C-Advanced), aggressive management of isolated extrahepatic disease is supported by the literature, where several studies report prolonged disease-free survival after resection [2,13-16]. Hwang et al. reported a 31% survival at two years in 23 patients undergoing resection of pulmonary metastases from HCC [13]. Many of these studies include patients undergoing surgery for LNM, though sample sizes are small. For instance, Ikegami et al. reported outcomes of six patients presenting with isolated LNM; two of four patients undergoing surgery survived for over four years [2].

HCC is radiosensitive; in the largest series of patients receiving palliative, conventionally-fractionated radiation for LNM, the objective response rate to radiation was 97% [17]. SBRT is thus an attractive alternative to surgery, especially when surgical challenges exist due to the celiac axis, portal vessels, inferior vena cava, and transplanted livers. The increasing prevalence of nonalcoholic fatty liver disease and associated metabolic syndrome may also make many patients poor surgical candidates [18]. Nonetheless, the high dose/fraction of SBRT and presence of nearby bowel necessitate rigorous consideration of patient instructions (e.g. empty stomach), motion and respiratory management, image guidance, and careful treatment planning to maintain safe duodenum and stomach doses [19]. These issues are known and well-described in the setting of pancreas SBRT [20]. Indeed, the challenge of balancing dose for tumor control and limiting toxicity is illustrated in cases A and B. Patient A received the lowest dose (BED 48 Gy) and did not have a very good response, but also had no significant toxicity. Patient B received a higher dose (BED 72 Gy), achieved a near-complete response, and is cancer-free, but experienced grade 3 duodenal toxicity. Despite careful treatment planning to meet duodenum dose constraints, V25 was relatively high at 41 cc (goal is typically <10 cc), and this likely contributed to the toxicity observed.

Patients with “oligometastatic” cancers are increasingly considered for aggressive locoregional treatment, with two recent high-profile randomized phase II trials suggesting a survival benefit [9,10]. Thus, it is important to establish whether long-term disease-free survival can safely be achieved in different disease settings such as recurrent HCC. Such benefit versus risk considerations are complex, especially for post-transplant patients. A comprehensive review and helpful multidisciplinary management algorithm was recently proposed by Au et al. [21]. Their article highlights pertinent clinical differences between disseminated and oligo-recurrent disease, as well as the importance of a multidisciplinary team of clinicians representing hepatology, transplant surgery, medical and radiation oncology, and diagnostic and interventional radiology.

## Conclusions

In conclusion, this series demonstrates favorable outcomes after SBRT for isolated HCC LNM. These results may be of interest to physicians across multiple specialties, given that optimal management of HCC requires close multidisciplinary collaboration. In centers with appropriate expertise, SBRT could be a valuable non-invasive treatment option for patients with LNM. SBRT may be particularly useful when surgical challenges exist (eg, celiac axis involvement) or when treatment options are limited by prior transplant.

## References

[1] Xia F, Wu L, Lau WY, Li G, Huan H, Qian C, Ma K, Bie P (2014). Positive lymph node metastasis has a marked impact on the long-term survival of patients with hepatocellular carcinoma with extrahepatic metastasis. PLoS One.

[2] Ikegami T, Yoshizumi T, Kawasaki J (2017). Surgical resection for lymph node metastasis after liver transplantation for hepatocellular carcinoma. Anticancer Res.

[3] Network NCC (2020). National Comprehensive Cancer Network, hepatobiliary cancers. https://www.nccn.org/professionals/physician_gls/pdf/hepatobiliary.pdf.

[4] Marrero JA, Kulik LM, Sirlin CB (2018). Diagnosis, staging, and management of hepatocellular carcinoma: 2018 practice guidance by the American Association for the Study of Liver Diseases. Hepatology.

[5] Wahl DR, Stenmark MH, Tao Y (2016). Outcomes after stereotactic body radiotherapy or radiofrequency ablation for hepatocellular carcinoma. J Clin Oncol.

[6] Shen PC, Chang WC, Lo CH (2019). Comparison of stereotactic body radiation therapy and transarterial chemoembolization for unresectable medium-sized hepatocellular carcinoma. Int J Radiat Oncol Biol Phys.

[7] Kim N, Kim HJ, Won JY (2019). Retrospective analysis of stereotactic body radiation therapy efficacy over radiofrequency ablation for hepatocellular carcinoma. Radiother Oncol.

[8] Eisenhauer EA, Therasse P, Bogaerts J (2009). New response evaluation criteria in solid tumours: revised RECIST guideline (version 1.1). Eur J Cancer.

[9] Gomez DR, Tang C, Zhang J (2019). Local consolidative therapy vs. maintenance therapy or observation for patients with oligometastatic non-small-cell lung cancer: long-term results of a multi-institutional, phase II, randomized study. J Clin Oncol.

[10] Palma DA, Olson R, Harrow S (2019). Stereotactic ablative radiotherapy versus standard of care palliative treatment in patients with oligometastatic cancers (SABR-COMET): a randomised, phase 2, open-label trial. Lancet.

[11] Jeng LB, Lee SG, Soin AS (2018). Efficacy and safety of everolimus with reduced tacrolimus in living-donor liver transplant recipients: 12-month results of a randomized multicenter study. Am J Transplant.

[12] Iavarone M, Invernizzi F, Czauderna C (2019). Preliminary experience on safety of regorafenib after sorafenib failure in recurrent hepatocellular carcinoma after liver transplantation. Am J Transplant.

[13] Hwang S, Kim YH, Kim DK (2012). Resection of pulmonary metastases from hepatocellular carcinoma following liver transplantation. World J Surg.

[14] Roayaie S, Schwartz JD, Sung MW (2004). Recurrence of hepatocellular carcinoma after liver transplant: patterns and prognosis. Liver Transpl.

[15] Bodzin AS, Lunsford KE, Markovic D, Harlander-Locke MP, Busuttil RW, Agopian VG (2017). Predicting mortality in patients developing recurrent hepatocellular carcinoma after liver transplantation: impact of treatment modality and recurrence characteristics. Ann Surg.

[16] Fernandez-Sevilla E, Allard MA, Selten J (2017). Recurrence of hepatocellular carcinoma after liver transplantation: is there a place for resection?. Liver Transpl.

[17] Zeng ZC, Tang ZY, Fan J (2005). Consideration of role of radiotherapy for lymph node metastases in patients with HCC: retrospective analysis for prognostic factors from 125 patients. Int J Radiat Oncol Biol Phys.

[18] Younossi Z, Anstee QM, Marietti M (2018). Global burden of NAFLD and NASH: trends, predictions, risk factors and prevention. Nat Rev Gastroenterol Hepatol.

[19] Pollom EL, Chin AL, Diehn M, Loo BW, Chang DT (2017). Normal tissue constraints for abdominal and thoracic stereotactic body radiotherapy. Semin Radiat Oncol.

[20] Oar A, Lee M, Le H (2020). Australasian Gastrointestinal Trials Group (AGITG) and Trans-Tasman Radiation Oncology Group (TROG) guidelines for pancreatic stereotactic body radiation therapy (SBRT). Pract Radiat Oncol.

[21] Au KP, Chok KS (2018). Multidisciplinary approach for post-liver transplant recurrence of hepatocellular carcinoma: a proposed management algorithm. World J Gastroenterol.

